# Effect of Endobronchial Valve Therapy on Pulmonary Perfusion and Ventilation Distribution

**DOI:** 10.1371/journal.pone.0118976

**Published:** 2015-03-30

**Authors:** Carmen Pizarro, Hojjat Ahmadzadehfar, Markus Essler, Izabela Tuleta, Rolf Fimmers, Georg Nickenig, Dirk Skowasch

**Affiliations:** 1 Department of Internal Medicine II, Cardiology, Pneumology and Angiology, University Hospital Bonn, Bonn, Germany; 2 Department of Nuclear Medicine, University Hospital Bonn, Bonn, Germany; 3 Institute for Medical Biometry, Informatics and Epidemiology, University Hospital Bonn, Bonn, Germany; University Hospital Freiburg, GERMANY

## Abstract

**Introduction:**

Endoscopic lung volume reduction (ELVR) is an emerging therapy for emphysematous COPD. However, any resulting changes in lung perfusion and ventilation remain undetermined. Here, we report ELVR-mediated adaptations in lung perfusion and ventilation, as investigated by means of pulmonary scintigraphy.

**Methods:**

In this observational study, we enrolled 26 patients (64.9±9.4 yrs, 57.7% male) with COPD heterogeneous emphysema undergoing ELVR with endobronchial valves (Zephyr, Pulmonx, Inc.). Mean baseline FEV1 and RV were 32.9% and 253.8% predicted, respectively. Lung scintigraphy was conducted prior to ELVR and eight weeks thereafter. Analyses of perfusion and ventilation shifts were performed and complemented by correlation analyses between paired zones.

**Results:**

After ELVR, target zone perfusion showed a mean relative reduction of 43.32% (p<0.001), which was associated with a significant decrease in target zone ventilation (p<0.001). Perfusion of the contralateral untreated zone and of the contralateral total lung exhibited significant increases post-ELVR (p = 0.002 and p = 0.005, respectively); both correlated significantly with the corresponding target zone perfusion adaptations. Likewise, changes in target zone ventilation correlated significantly with ventilatory changes in the contralateral untreated zone and the total contralateral lung (Pearson’s r: −0.42, p = 0.04 and Pearson’s r: −0.42, p = 0.03, respectively). These effects were observed in case of clinical responsiveness to ELVR, as assessed by changes in the six-minute walk test distance.

**Discussion:**

ELVR induces a relevant decrease in perfusion and ventilation of the treated zone with compensatory perfusional and ventilatory redistribution to the contralateral lung, primarily to the non-concordant, contralateral zone.

## Introduction

Chronic obstructive pulmonary disease (COPD) is a major cause of morbidity and mortality with an estimated worldwide prevalence of 10% in adults [1]. Given its progressive, wasting and frequently deadly course [2], efforts have been made over the past few years to complement the established medical approaches of inhaler therapy, supplemental long-term oxygen therapy, smoking cessation and comprehensive pulmonary rehabilitation. These procedures primarily address the emphysematous destruction of lung parenchyma defined by permanent enlargement of distal airspaces and loss of elastic retraction. Lung volume reduction surgery as a therapeutic modality for severe emphysema reduces the mismatch in size between the hyperinflated lungs and the chest cavity [3]. It thereby restores the efficiency of respiratory muscle activity, decreases the work of breathing and alleviates dyspnoea. In consideration of its significant perioperative mortality and cost [4, 5], non-surgical bronchoscopic means of achieving lung volume reduction have been developed. These procedures comprise endobronchial placement of one-way valves, coils, biological sealants and thermal ablation. However, endobronchial valve (EBV) therapy has the most clinical experience and publication history. The Endobronchial Valve for Emphysema Palliation Trial (VENT) evaluated unilateral treatment with endobronchial valves in patients with severe heterogeneous emphysema. The patients were randomly assigned to receive either endoscopic placement of Zephyr endobronchial valves or standard medical care [6]. The study concluded that EBV placement resulted in modest positive effects on expiratory flow rates, exercise tolerance and quality of life. The European VENT study cohort demonstrated that the presence of complete fissure, that in turn is suggestive of a lack of collateral ventilation, was associated with a superior clinical result. It indicates that collateral ventilation from adjacent lobes limits the clinical response to EBV implantation [7].

From a mechanical point of view, endobronchial valves allow air and mucus to exit the treated area, but prevent air from re-entering the occluded segment [8]. The intention of EBV therapy is a reduction up to complete atelectasis of the hyperinflated lobe distal to the implanted valves. Whereas volumetric post-procedural changes—identified by CT-imaging—have frequently been described [9, 10], the simultaneously provoked regional hypoxemia and its thereby presumably induced hypoxic vasoconstriction have mostly been disregarded.

The aim of the present study was to analyze by use of pulmonary scintigraphy the ventilatory and perfusional shifts following endoscopic lung volume reduction (ELVR) and to correlate the results with the clinical outcome measures.

## Methods

### Study population

Of thirty-five screened patients, twenty-six patients aged ≥ 18 years with advanced, emphysematous COPD and referred to the University Hospital Bonn (Bonn, Germany) for elective ELVR by EBV were confirmed eligible for this observational study. The study was undertaken during the period from November 2012 to December 2013. The inclusion criteria corresponded to the VENT study’s criteria [6] and comprised heterogeneity of emphysema, a forced expiratory volume in one second (FEV1) of 15 to 45% of predicted, a residual volume (RV) > 150% of predicted, a total lung capacity (TLC) > 100% of predicted and a six-minute walk test distance (6MWTD) ≥ 140 m. Additionally, pre- and post-procedural pulmonary scintigraphy was required. In accordance with the VENT study [6], we excluded patients presenting: an elevated pulmonary exacerbation rate (≥ 2 exacerbations per year), continued nicotine consumption, an underlying alpha-1 antitrypsin deficiency, pulmonary giant bullae or advanced cardiac comorbidities.

### Ethics statement

The study was approved by the University Hospital of Bonn medical ethics committee and all patients gave their written informed consent. All data were analysed anonymously. The study was performed in accordance with the principles expressed in the Declaration of Helsinki

### Study design

Thoracic computed tomography was used to characterize the emphysematous distribution and assess heterogeneity. The target lobe was defined by considering the visual estimation of the highest degree of emphysematous destruction and heterogeneity.

All patients underwent a detailed clinical history and physical examination. The 6MWT was performed the day prior to ELVR and eight weeks thereafter to determine clinical responder status to ELVR therapy, with a change in 6MWTD ≥ 25 m indicating clinical responsiveness [11]. Additionally, pulmonary function tests and the COPD Assessment Test (CAT) were used pre- and post-procedurally to quantify the impact of COPD symptoms on patients´ health status [12]. The occurrence of pulmonary atelectasis was determined by chest X-ray eight weeks post-ELVR.

### Endobronchial valve implantation

Zephyr (Pulmonx, Inc., Redwood City, Calif., USA) endobronchial valves were used in this study. They consist of a nitinol skeleton with a silicone body and a mounted one-way valve [13, 14]. We performed flexible bronchoscopy and the Chartis Pulmonary Assessment System (Pulmonx, Inc., Redwood City, Calif., USA) to evaluate collateral ventilation (CV) [15]. In the absence of CV, we proceeded to valve placement. Bronchoscopy was conducted under moderate sedation by use of the short-acting benzodiazepine midazolam and low dose propofol, allowing for spontaneous breathing. All bronchoscopies were performed by the same experienced bronchoscopist (D.S.) and did not require anaesthesiological assistance.

### V/Q lung scintigraphy

**Fig 1:**
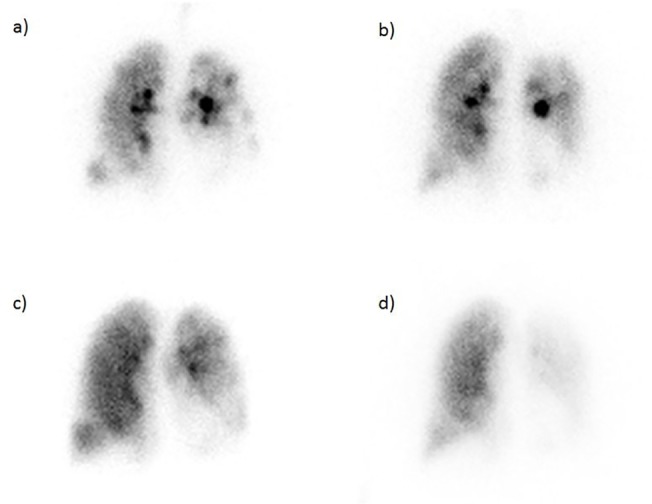
Pulmonary scintigraphy with ventilatory (a) pre-procedural, b) post-procedural) and perfusional (c) pre-procedural, d) post-procedural) imaging in a left upper lobe treated patient.

Ventilation/perfusion (V/Q) scintigraphy scans were performed pre and 8-week post ELVR therapy (Fig. 1). The ventilation scan was carried out by acquisition of planar images after inhalation of Technegas (Cyclomedica, New South Wales, Australia), an ultra-fine dispersion of Technetium-99m labelled carbon. For perfusion scintigraphy, macro-aggregated albumin, radiolabelled with Technetium-99m (99mTC-MAA) and having an administered activity of 200 MBq, was intravenously injected and followed by planar imaging. The division of each lung into zonal thirds was used to define the targeted zone as the treated third and to compare the targeted with the non-targeted ipsilateral lung, defined as the remaining two thirds.

### Statistical analysis

The zonal baseline and post-treatment percentage changes in total lung perfusion and ventilation were evaluated in all subjects. Descriptive statistics are presented as means (±SD) or medians (range) when appropriate; the ELVR-induced scintigraphic changes are presented as means (±SD), means (95% confidence interval) or relative intra-zonal percentages of baseline values. Paired t-tests were used to compare results before and after EBV placement. A p-value <0.05 was considered the threshold for statistical significance. Statistical dependence between inter-zonal perfusional and ventilatory shifts was assessed by Pearson correlation coefficient. The unpaired t-test was performed to assess scintigraphic changes as a function of responder status. All statistical analyses were performed using SPSS Statistics 22 software (IBM, Armonk, NY, USA).

## Results

The pre-procedural patient demographic characteristics and clinical data are summarised in Table 1. The baseline pulmonary function tests revealed a mean FEV1 of 0.93 l (±0.26 l) in absolute terms, or 32.89% (±6.21%) of the predicted value. The residual volume (RV) averaged 5.75 l (±1.12 l) or 253.76% (±47.45%) of predicted; the RV/TLC-ratio was 75.26% (±6.31%) or 188.94% (±22.67%) of predicted. The lobar treatment percentages for the right upper lobe, right lower lobe, left upper lobe and left lower lobe were 34.6%, 19.2%, 15.4% and 30.8%, respectively. The mean number of valves placed per lobe accounted for 4.2.

**Table 1:** Patient demographic and functional characteristics (26 patients).

Age, years	64.9 ± 9.4
Mal gender, %	57.7
Smoking history, pack-years	38.0 ± 15.2
FEV1, l	0.93 ± 0.26
FEV1, % predicted	32.89 ± 6.21
RV, l	5.75 ± 1.12
RV, % predicted	253.76 ± 47.45
RV/TLC ratio	75.26 ± 6.31
RV/TLC ratio, % predicted	188.94 ± 22.67
DLCO, mmol/min/kPa	2.37 ± 1.13
DLCO, % predicted	28.75 ± 12.64
PaO_2_, mmHg	57.8 ± 2.1
PaCO_2_, mmHg	43.8 ± 1.9
CAT	23.9 ± 7.3
6MWTD, m	310 (140–600)
Target lobe distribution, %	
Right upper lobe	34.6
Left upper lobe	15.4
Right lower lobe	19.2
Left lower lobe	30.8
Baseline perfusion per target zone, %	
Right upper zone	10.4±4.8
Left upper zone	12.3±5.5
Right lower zone	18.3±10.4
Left lower zone	7.9±3.8
Medication use, %	
Long-acting beta agonist	100
Long-acting anticholinergics	100
Inhaled glucocorticoids	96.2
Triple inhaler therapy	96.2
Systemic glucocorticoids	7.7
PDE-4 inhibitor (Roflumilast)	46.2
Long-term oxygen therapy	46.2

Data are presented as percentages, mean ± SD or median and range (in parentheses).Abbreviations:

FEV1 = forced expiratory volume in 1 second

RV = residual volume

RV/TLC = residual volume/total lung capacity

DL_CO_ = diffusion capacity for carbon monoxide

PaO_2_ = arterial oxygen partial pressure

PaCO_2_ = arterial carbon dioxide partial pressure

CAT = COPD Assessment Test

6MWTD = 6 minute walk test distance

**Fig 2:**
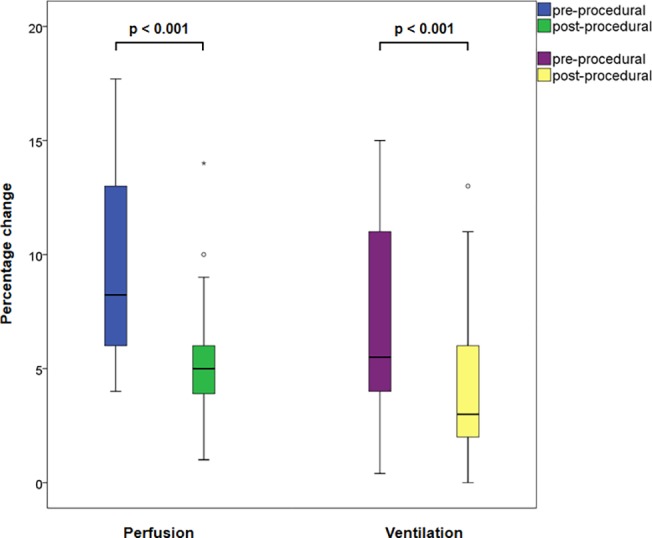
Boxplot of ELVR-mediated changes in target zone perfusion and ventilation.

A comparison of the perfusion of the treated zone prior to and after ELVR revealed a significant absolute perfusional reduction of 3.86% (95% CI: −5.10–−2.61; p<0.001), corresponding to a 43.32% relative decrease (Fig. 2). Perfusion in the ipsilateral, non-targeted zone exhibited a non-significant mean increase of 0.61% (95% CI: −1.40–+2.61; p = 0.54). Concurrently, perfusion of the contralateral whole lung increased significantly by 5.96% and 3.26% (95% CI: +1.11–+5.41; p = 0.005) in relative and absolute terms; likewise, the contralateral, non-concordant zone revealed significant perfusional gain (a relative and absolute increase of 6.78% and 2.72%, respectively; 95% CI: +1.11–+4.34; p = 0.002). Perfusion in the remaining zones did not exhibit significant shifts (Table 2).

**Table 2:** Change in perfusion of single zones from baseline to eight weeks after EBV treatment.

	Baseline	Week 8	Absolute change in perfusion (95% CI)	Relative change in perfusion	p-value
Target zone	8.91 ± 3.77	5.06 ± 2.76	−3.86 (−5.10–−2.61)	−43.32%	<0.001
Ipsilateral, nontargeted zone	36.39 ± 7.98	36.99 ± 9.58	+0.61 (−1.40–+2.61)	+1.68%	0.54
Total ipsilateral lung	45.29 ± 9.44	42.82 ± 10.99	−2.47 (−5.06–+0.11)	−5.45%	0.06
Contralateral, concordant zone	14.23 ± 5.57	15.10 ± 6.55	+0.87 (−1.01–+2.74)	+6.11%	0.35
Contralateral, non-concordant zone	40.09 ± 7.46	42.81 ± 8.59	+2.72 (+1.11–+4.34)	+6.78%	<0.01
Total contralateral lung	54.70 ± 9.44	57.95 ± 10.42	+3.26 (+1.11–+5.41)	+5.96%	<0.01

Data are presented as mean percentage (±SD) of whole lung perfusion, absolute intrazonal percentage change and 95% Confidence Interval (in parentheses) or relative intrazonal percentage change from baseline value.

Analogously, ventilation in the target zone decreased significantly by an absolute 3.01% (95% CI: −4.46–−1.56; p<0.001) and a relative 42.22%, whereas there were no significant differences in ventilatory changes in the other zones (Table 3).

**Table 3:** Change in ventilation of single zones from baseline to eight weeks after EBV treatment.

	Baseline	Week 8	Absolute change in perfusion (95% CI)	Relative change in ventilation	p-value
Target zone	7.13 ± 4.40	4.12 ± 3.45	−3.01 (−4.46–−1.56)	−42.22%	<0.001
Ipsilateral, nontargeted zone	39.09 ± 7.14	40.39 ± 7.79	+1.30 (−0.83–+3.42)	+3.33%	0.22
Total ipsilateral lung	46.27 ± 6.90	44.48 ± 8.50	−1.79 (−4.06–+0.49)	−3.87%	0.12
Contralateral, concordant zone	13.53 ± 6.08	13.75 ± 6.43	+0.22 (−1.78–+2.21)	+1.63%	0.83
Contralateral, non-concordant zone	40.16 ± 6.17	41.51 ± 8.89	+1.35 (−0.84–+3.54)	+3.36%	0.22
Total contralateral lung	53.71 ± 6.88	55.59 ± 8.47	+1.88 (−0.38–+4.15)	+3.50%	0.10

Data are presented as mean percentage (±SD) of whole lung perfusion, absolute intrazonal percentage change and 95% Confidence Interval (in parentheses) or relative intrazonal percentage change from baseline value.

Correlation analysis by Pearson correlation coefficient (Pearson´s r) between paired zones evinced significant dependence between perfusional adaptations in the target zone, on the one hand, and the untreated contralateral zone and total contralateral lung, on the other hand (Pearson´s r: −0.40, p = 0.04 and Pearson´s r: −0.42, p = 0.03, respectively). Analogically, ventilatory decrease in the treated zone showed significant redistribution to the contralateral non-treated zone and to the total contralateral lung (lung (Pearson´s r: −0.42, p = 0.04 and Pearson´s r: −0.42, p = 0.03, respectively).

Differentiation dependent on upper versus lower lobe treatment revealed a 1.8-fold greater decline of target zone perfusion in the upper lobe treated collectively (p = 0.06); changes in ipsilateral total lung perfusion reached significance (p = 0.03) in favour of the upper lobe targeted patients.

At eight weeks, pulmonary function testing revealed an absolute and relative increase in FEV1 by 5.17% and 15.72% of the predicted value, respectively (95%CI: +0.53%–+9.81; p = 0.03). The RV/TLC ratio that has been shown to strongly correlate with the degree of airways obstruction [16], decreased absolutely by 12.53% of predicted (95%CI: −22.42–−2.64; p = 0.02).

Chest X-rays at week 8 revealed that post-procedural complete atelectasis with lobar exclusion occurred in 53.8% of treated patients. A subdivision of the complete cohort by the presence or absence of atelectasis evinced a significant gain in FEV1 of predicted by 6.84% and 19.79% (p = 0.02)—in absolute and relative terms, respectively—in the case of atelectasis, whereas the absence of atelectasis implicated non-significant changes in pulmonary function values.

A responder analysis based upon the 6MWT revealed a significant post-therapy increase in the 6MWTD of 61.0 m (±106.9 m, p = 0.01). 18/26, i.e., 69.2% of treated patients were clinical responders. Clinical responsiveness was associated with the perfusional and ventilatory changes described above; non-responders showed only a significant reduction in target zone perfusion (p = 0.005).

Mean pre- and post-procedural CAT scores were 23.9±7.3 and 22.4±6.5, respectively; thus the minimal important difference, recently estimated to account for 2 points [17], was not met.

Overall, complication rate was low. There were no serious intra-procedural complications. Post-procedurally, exacerbations of COPD occurred in 15.4% of patients (n = 4) and resolved with antibiotic and steroidal therapy, without necessity for valve removal. No pneumonia distal to the valves or hemoptysis were observed. Two patients exhibited post-procedural pneumothorax; both of them were managed successfully by temporary chest tube implantation.

## Discussion

The current study characterizes scintigraphic features pre- and post-ELVR and demonstrates an ELVR-induced interdependency of perfusional and ventilatory shifts.

The bronchoscopic treatment of emphysema as an emerging therapeutic modality has engendered a new platform for assessing the emphysematous physiology of the lung. The rationale for ELVR comprehends several theoretic considerations. First of all, the reduction of overinflated pulmonary regions is considered to mitigate the mechanical overstretch of the respiratory muscles. It leads to an improvement in diaphragm mechanics and alleviation of the work of breathing [18, 19]. Another consideration is based on the assumption that less emphysematously destroyed lung has more elastic retraction [20]. It offers an increase in expiratory airflow by pulling on the bronchioles. Finally, the correction of regional discrepancies in ventilation and perfusion should optimise alveolar gas exchange and alleviate hypoxemia.

In accordance with the VENT study [6], we defined the target lung lobe by computed tomography-assessed distribution and heterogeneity of emphysema. The value of a perfusion scintigraphy in the selection of patients for surgical lung volume reduction was studied by Chandra et al. [21]. In contrast to the mere anatomical assessment of emphysema provided by CT, he attributed to pre-procedural lung perfusion additional diagnostic value through its reflection of regional lung function. Following this line of thought, LVR—surgically or endoscopically executed—might even diminish post-procedural perfusion as a reflection of the LVR-mediated reduction of regional lung function. Reduced perfusion as a result of endobronchial occlusion, e.g., by endobronchial valve placement, requires on the other hand a prior decrease in ventilation in terms of hypoxic pulmonary vasoconstriction [22].

Chung and colleagues examined serial changes in ventilation and perfusion at baseline, at day 30 and at day 90 post-ELVR in six patients undergoing EBV-implantations that were exclusively performed in the left upper lobe [23]. Post-ELVR measurements revealed reductions in ventilation and perfusion in the left upper zone, accompanied by ventilatory and perfusion increases in the right lower zone. In consideration of the small sample size of six patients and the left upper lobe being the sole targeted lobe, we expanded the study population to allow examination of different targets. The corresponding percentages were 34.6%, 19.2%, 15.4% and 30.8% for the right upper lobe, right lower lobe, left upper lobe and left lower lobe, respectively. Irrespective of the treated lobe, its perfusion and ventilation decreased significantly and showed a consistent redistributive pattern. We extrapolated Chung’s finding of perfusional gain in the right lower zone and whole right lung to a generalization of significant perfusional increase in the contralateral non-concordant and total lung, showing statistical significance in correspondence analysis with the target zone’s perfusion adaptations that implies an ELVR-mediated perfusional redistribution primarily to the contralateral lung. Nonetheless, Chung’s report of reduced ventilation in the ipsilateral, non-treated lung differed from our observation of an increase in perfusion and ventilation, the latter being concordant with the ELVR-implied concept of gain in function of the healthier lung.

We proceeded to EBV placement after excluding evidence of collateral ventilation (CV) by use of the Chartis System (Pulmonx, Inc., Redwood City, Calif., USA), an endobronchial catheter system that measures pressures and flows during respiration and calculates the resistance of the collateral channels [15]. Its accuracy in assessing CV was studied by Herth et al. [24]. They defined a computed tomography-measured, EBV-mediated reduction of target lobe volume ≥ 350 ml to be significant. Chartis revealed a reliability of 75% in predicting significant volume reduction, reflecting efficiency in estimating CV. In our study, the post-ELVR target zone’s ventilation and perfusion was reduced significantly, indicating the accuracy of the previous Chartis-guided CV-evaluation. Nonetheless, in a small subset of patients CV-measurement was inconclusive, primarily due to multiple mucous plugs, resulting in the observed reduction of complete atelectasis frequency that did not alter occurrence, but presumably extent of ELVR-mediated scintigraphic changes.

We determined the clinical responsiveness to ELVR by improvement in the 6MWTD. The minimal important difference for the 6MWTD in COPD was defined by Holland and his colleagues to be 25 m, the absolute 6MWTD-changes being more sensitive than the relative changes from baseline value [11], and was applied by Argula et al. to evaluate responder status to EBV therapy [25]. In our sample, we observed a significant improvement in the 6MWTD of 61.0 ± 106.9 m (95% CI: +17.8–+104.3; p = 0.01), with 18 out of 26 patients being clinical responders. The presently exhibited scintigraphic shifts were observed in case of clinical responsiveness. Brown et al measured computed tomographic changes following EBV-implantation and ascertained volumetric shifts to contralateral lung and non-treated ipsilateral lobe [10]. In our study, we targeted the lobe offering the highest degree of emphysematous damage. In view of the above described volumetric adaptations and the presently observed perfusional redistribution to healthier lung areas with preserved functional reserve, ELVR-mediated redistributive extent determines amelioration of exercise tolerance, quantified by 6MWT.

In a retrospective analysis of the VENT trial treatment group, Argula et al. recently described a low baseline target perfusion to be a predictor of superior EBV therapy outcome, as determined by the 6MWTD [25]. They stratified low and high perfusion at the median percent perfusion for each target zone. After applying their median-driven perfusion dichotomy to our study population, improvement in the 6MWTD was independent of low or high baseline target perfusion.

There are several limitations of this study to be considered. The study design was observational without prior patient randomisation, blinding, or inclusion of a CV-positive control group. Given the elevated radiation exposure that encompasses scintigraphic imaging, our study sample size was modest. However, it exceeds the population studied by Chung et al. and allows extrapolating their results to different target lobes.

A further limitation was the deduction of the lung zones by craniocaudally dividing each lung into lobar zones. This carries the potential risk of measuring adjacent lobar expansion post-ELVR rather than through anatomically defined lobes. Sciurba et al. described an EBV-induced, HRCT-evaluated volume reduction in the target lobe that was associated with a volume expansion of the adjacent non-targeted lobe [6]. In view of the above, and considering that our study population manifested a non-significant perfusion and ventilation increase in the ipsilateral untreated lung, a mere scintigraphic pulmonary division might underestimate the post-interventional adjacent lung’s gain in perfusion and ventilation. Therefore, a post-procedural CT-guided volumetric assessment would be a valuable complement to our scintigraphic observations and to pre-procedural CT-guided evaluation of contralateral lung´s emphysematous affection and functional reserves.

In conclusion, EBV therapy induces relevant decreases in target zone ventilation and perfusion that are accompanied by perfusion and ventilation redistribution to the contralateral lung. Future studies in larger populations with prospective evaluations are warranted to clarify the significance of this finding.

## References

[1] Rabe KF, Hurd S, Anzueto A, Barnes PJ, Buist SA, Calverley P, et al (2007) Global Initiative for Chronic Obstructive Lung Disease. Global strategy for the diagnosis, management, and prevention of chronic obstructive pulmonary disease: GOLD executive summary. Am J Respir Crit Care Med 176: 532–555. 17507545 10.1164/rccm.200703-456SO

[2] Mannino DM, Buist AS (2007) Global burden of COPD: risk factors, prevalence, and future trends. Lancet 370: 765–773. 17765526 10.1016/S0140-6736(07)61380-4

[3] Martinez FJ, Chang A (2005) Surgical therapy for chronic obstructive pulmonary disease. Semin Respir Crit Care Med 26: 167–191. 16088435 10.1055/s-2005-869537

[4] Fishman A, Martinez F, Naunheim K, Piantadosi S, Wise R, Ries A, et al (2003) A randomized trial comparing lung-volume-reduction surgery with medical therapy for severe emphysema. N Engl J Med 348: 2059–2073. 12759479 10.1056/NEJMoa030287

[5] Naunheim KS, Wood DE, Mohsenifar Z, Sternberg AL, Criner GJ, DeCamp MM, et al (2006) Long-term follow-up of patients receiving lung-volume-reduction surgery versus medical therapy for severe emphysema by the National Emphysema Treatment Trial Research Group. Ann Thorac Surg 82: 431–443. 16888872 10.1016/j.athoracsur.2006.05.069

[6] Sciurba FC, Ernst A, Herth FJ, Strange C, Criner GJ, Marquette CH, et al (2010) A randomized study of endobronchial valves for advanced emphysema. N Engl J Med 363: 1233–1244. 10.1056/NEJMoa0900928 20860505

[7] Herth FJ, Noppen M, Valipour A, Leroy S, Vergnon JM, Ficker JH, et al (2012) Efficacy predictors of lung volume reduction with Zephyr valves in a European cohort. Eur Respir J 39: 1334–1342. 10.1183/09031936.00161611 22282552

[8] Wan IY, Toma TP, Geddes DM, Snell G, Williams T, Venuta F, et al (2006) Bronchoscopic lung volume reduction for end-stage emphysema: report on the first 98 patients. Chest 129: 518–526. 16537847 10.1378/chest.129.3.518

[9] D'Andrilli A, Vismara L, Rolla M, Ibrahim M, Venuta F, Pochesci I, et al (2009) Computed tomography with volume rendering for the evaluation of parenchymal hyperinflation after bronchoscopic lung volume reduction. Eur J Cardiothorac Surg 35: 403–407. 10.1016/j.ejcts.2008.10.045 19084426

[10] Brown MS, Kim HJ, Abtin FG, Strange C, Galperin-Aizenberg M, Pais R, et al (2012) Emphysema lung lobe volume reduction: effects on the ipsilateral and contralateral lobes. Eur Radiol 22: 1547–1555. 10.1007/s00330-012-2393-6 22466511

[11] Holland AE, Hill CJ, Rasekaba T, Lee A, Naughton MT, McDonald CF (2010) Updating the minimal important difference for six-minute walk distance in patients with chronic obstructive pulmonary disease. Arch Phys Med Rehabil 91: 221–225. 10.1016/j.apmr.2009.10.017 20159125

[12] Jones PW, Harding G, Berry P, Wiklund I, Chen WH, Kline Leidy N (2009) Development and first validation of the COPD Assessment Test. Eur Respir J 34: 648–654. 10.1183/09031936.00102509 19720809

[13] Toma TP, Hopkinson NS, Hillier J, Hansell DM, Morgan C, Goldstraw PG, et al (2003) Bronchoscopic volume reduction with valve implants in patients with severe emphysema. Lancet 361: 931–933. 12648974 10.1016/S0140-6736(03)12762-6

[14] Venuta F, de Giacomo T, Rendina EA, Ciccone AM, Diso D, Perrone A, et al (2005) Bronchoscopic lung-volume reduction with one-way valves in patients with heterogenous emphysema. Ann Thorac Surg 79: 411–416. 15680805 10.1016/j.athoracsur.2004.07.048

[15] Mantri S, Macaraeg C, Shetty S, Aljuri N, Freitag L, Herth F, et al (2009) Technical advances: measurement of collateral flow in the lung with a dedicated endobronchial catheter system. J Bronchology Interv Pulmonol 16: 141–144. 10.1097/LBR.0b013e3181a40a3e 23168520

[16] Dykstra BJ, Scanlon PD, Kester MM, Beck KC, Enright PL (1999) Lung volumes in 4,774 patients with obstructive lung disease. Chest 115: 68–74. 9925064 10.1378/chest.115.1.68

[17] Kon SS, Canavan JL, Jones SE, Nolan CM, Clark AL, Dickson MJ, et al (2014) Minimally clinically important difference for the COPD Assessment Test: a prospective analysis. Lancet Respir Med 1: 195–203. 10.1016/S2213-2600(14)70001-3 24621681

[18] O'Donnell DE, Webb KA (2008) The major limitation to exercise performance in COPD is dynamic hyperinflation. J Appl Physiol 105: 753–755. 10.1152/japplphysiol.90336.2008b 18678624

[19] Casanova C, Cote C, de Torres JP, Aguirre-Jaime A, Marin JM, Pinto-Plata V, et al (2005) Inspiratory-to-total lung capacity ratio predicts mortality in patients with chronic obstructive pulmonary disease. Am J Respir Crit Care Med 171: 591–597. 15591470 10.1164/rccm.200407-867OC

[20] Martin C, Frija J, Burgel PR (2013) Dysfunctional lung anatomy and small airways degeneration in COPD. Int J Chron Obstruct Pulmon Dis 8: 7–13. 10.2147/COPD.S28290 23319856 PMC3540907

[21] Chandra D, Lipson DA, Hoffman EA, Hansen-Flaschen J, Sciurba FC, Decamp MM, et al (2010) Perfusion scintigraphy and patient selection for lung volume reduction surgery. Am J Respir Crit Care Med 182: 937–946. 10.1164/rccm.201001-0043OC 20538961 PMC2970864

[22] Sommer N, Dietrich A, Schermuly RT, Ghofrani HA, Gudermann T, Schulz R, et al (2008) Regulation of hypoxic pulmonary vasoconstriction: basic mechanisms. Eur Respir J 32: 1639–1651. 10.1183/09031936.00013908 19043010

[23] Chung SC, Peters MJ, Chen S, Emmett L, Ing AJ (2010) Effect of unilateral endobronchial valve insertion on pulmonary ventilation and perfusion: a pilot study. Respirology 15: 1079–1083. 10.1111/j.1440-1843.2010.01815.x 20636308

[24] Herth FJ, Eberhardt R, Gompelmann D, Ficker JH, Wagner M, Ek L, et al (2013) Radiological and clinical outcomes of using Chartis to plan endobronchial valve treatment. Eur Respir J 41: 302–308. 10.1183/09031936.00015312 22556025

[25] Argula RG, Strange C, Ramakrishnan V, Goldin J (2013) Baseline regional perfusion impacts exercise response to endobronchial valve therapy in advanced pulmonary emphysema. Chest 144: 1578–1586. 10.1378/chest.12-2826 23828481

